# Ultra-high-performance liquid chromatography-tandem mass spectrometry analysis of serum metabolomic characteristics in people with different vitamin D levels

**DOI:** 10.1515/med-2023-0658

**Published:** 2023-03-01

**Authors:** Huan Li, Xiaomin Xie, Li Zhang, Yanting He, Huili Liu, Dan Qiang, Guirong Bai, Ling Li, Yanpan Tang

**Affiliations:** Department of Endocrinology, The First People’s Hospital of Yinchuan, Yinchuan City, 750001, Ningxia Hui Autonomous Region, China; Department of Endocrinology, The First People’s Hospital of Yinchuan, Yinchuan City, Liqun West Street 2, 750001, Ningxia Hui Autonomous Region, China

**Keywords:** metabolomics, ultra-high-performance liquid chromatography-tandem mass spectrometry, vitamin D, cholesterol metabolism

## Abstract

Vitamin D is a fat-soluble vitamin with multiple functions. However, the metabolism of people with different vitamin D concentrations is still unclear. Herein, we collected clinical data and analysed the serum metabolome of people with 25-hydroxyvitamin D (25[OH]D) ≥40 ng/mL (A), 30 ng/mL ≤25(OH)D <40 ng/mL (B) and 25(OH)D <30 ng/mL (C) by the ultra-high-performance liquid chromatography-tandem mass spectrometry method. We found that haemoglobin A1c, fasting blood glucose, fasting insulin, homeostasis model assessment of insulin resistance and thioredoxin interaction protein were enhanced, while HOMA-β was reduced with the decrease of 25(OH)D concentration. In addition, people in the C group were diagnosed with prediabetes or diabetes. Metabolomics analysis showed that seven, thirty-four and nine differential metabolites were identified in the groups B vs A, C vs A and C vs B, respectively. Metabolites associated with cholesterol metabolism and bile acid biosynthesis, such as 7-ketolithocholic acid, 12-ketolithocholic acid, apocholic acid, *N*-arachidene glycine and d-mannose 6-phosphate, were significantly upregulated in the C group compared with the A or B groups. In conclusion, the disorder of vitamin D metabolism may be related to cholesterol metabolism and bile acid biosynthesis. This study provided a basis for exploring the possible mechanism leading to abnormal vitamin D metabolism.

## 1 Introduction

Vitamin D is a fat-soluble vitamin and performs various functions in the body. Vitamin D is present in the body in two primary forms, and It is mainly produced in the skin following exposure to ultraviolet irradiation. The vitamin D is also absorbed via food intake. In the liver, vitamin D is hydroxylated by the enzyme 25-hydroxylase to form 25-hydroxyvitamin D [25(OH)D], which is subsequently hydroxylated to 1α,25-dihydroxyvitamin D [1α,25(OH)_2_D] [1]. Active vitamin D exerts its biological function by combining it with the vitamin D receptor (VDR). The vitamin D status is evaluated by detecting 25(OH)D serum concentration. In 2011, the Endocrine Society defined 25(OH)D less than 20 ng/mL as vitamin D deficiency, 25(OH)D more than 30 ng/mL as vitamin D sufficiency and 25(OH)D between 20 and 30 ng/mL as vitamin D insufficiency [2]. Studies have shown that high and low vitamin D levels are associated with various diseases, including chronic complications of type 2 diabetes, non-alcoholic fatty liver disease, obesity, anxiety and depression [3,4,5,6]. Excessive vitamin D may result in hypercalcaemia, hypercalciuria and hyperphosphatemia [7]. Vitamin D deficiency is presumed to participate in the pathogenesis of metabolic syndromes by promoting inflammatory response, increasing insulin resistance, promoting adipocyte differentiation and affecting lipid metabolism [8]. Some studies have indicated that vitamin D deficiency is related to type I and type II diabetes [9,10]. Interestingly, 1α,25(OH)2D plays a crucial role in glucose homeostasis through various mechanisms. It not only boosts the insulin sensitivity of target cells (skeletal muscle, liver and adipose tissue), but also directly protects β cells from harmful immune attacks [11,12]. However, the metabolic characteristics of people with different vitamin D levels are unclear.

Metabolomics technology combines analytical chemistry based on physics, stoichiometry based on mathematical computational modelling and life science based on biochemistry. The metabolomic techniques frequently used mainly include nuclear magnetic resonance, liquid chromatography and liquid chromatography-tandem mass spectrometry (LC-MS/MS) [13]. LC-MS/MS has been widely employed due to its advantages of high sensitivity, strong specificity and simple sample treatment [14]. Ultra-high-performance LC-MS/MS (UPLC-MS/MS) has high separation efficiency and sufficient resolution. UPLC-MS/MS is a powerful method for determining metabolomics divergence derived from disease-related changes [15,16]. Therefore, UPLC-MS/MS could help analyse the metabolomics of people with different vitamin D levels.

In the present study, we aimed to investigate the metabolomic changes in people with different vitamin D levels. We assessed the clinical characteristics and the metabolites measured with UPLC-MS/MS in a cohort stratified on the full range of vitamin D levels. Moreover, we explored the possible mechanism leading to abnormal vitamin D metabolism.

## 2 Materials and methods

### 2.1 Subjects

Forty subjects were recruited from the physical examination Centre of The First People’s Hospital of Yinchuan. There were 22 males and 18 females, aged 27 to 57. According to 25(OH)D levels, they were divided into three groups: A, 15 people with 25(OH)D ≥40 ng/mL; B, 15 people with 25(OH)D between 30–40 ng/mL; C, 10 people with 25(OH)D <30 ng/mL. The disorder of vitamin D metabolism was defined as 25(OH)VD <30 ng/mL. None of the people received any lifestyle interventions or medications. Exclusion criteria are as follows: (a) prior diagnosis of prediabetes and diabetes; (b) patients with kidney, liver, or cancer; (c) patients with acute/chronic inflammatory disease; (d) patients with a history of cardiovascular and cerebrovascular diseases; (e) patients with thyroid dysfunction; (f) patients with blood disease; (g) people with alcohol or drug abuse or smoking; (h) women with hormone replacements; (i) patients who had taken vitamin D supplements in the last three months.


**Ethical approval:** This study was approved by the Ethics Committee of The First People’s Hospital of Yinchuan.
**Informed consent statement:** Written informed consent was obtained from all the participants.

### 2.2 General measurements

All subjects were given a unified questionnaire, and general data were collected, including gender, age, smoking history, height, weight, waist circumference (WC), hip circumference, body index (BMI), waist-hip ratio (WHR), systolic blood pressure (SBP) and diastolic blood pressure (DBP). All subjects were sampled from August 2020 to November 2020, and peripheral venous blood was collected after fasting for 8–12 h. The serum samples of all subjects were stored at −80°C. Fasting blood glucose (FBG), total cholesterol (TC), triglyceride (TG), low-density lipoprotein (LDL), high-density lipoprotein (HDL), uric acid (UA), urea, creatinine (Cr), alanine aminotransferase (ALT), aspartate aminotransferase (AST) and gamma-glutamyl transpeptidase (GGT) were measured using Beckman Coulter automatic biochemical analyser AU5821. The concentration of fasting insulin (FINS), haemoglobin A1c (HbA1c), 25(OH)D and thioredoxin-interacting protein (TXNIP) was determined by ELISA. Homeostasis model assessment of insulin resistance (HOMA-IR) = FBG*FINS/22.5. Homeostasis model assessment-β (HOMA-β) = 20*FINS/(FBG-3.5).

### 2.3 UPLC-MS/MS

A UPLC HSS T3 column (1.8 μm particle size, 2.1 mm × 100 mm) served as the stationary phase for the chromatographic isolation. The samples were isolated using acetonitrile and water (0.1% formic acid) through gradient elution with a flow rate of 0.4 mL/min. The gradient elution was as follows: 0 min, water/acetonitrile (95:5 V/V); 11.0 min, water/acetonitrile (10:90 V/V); 12.0 min, water/acetonitrile (10:90 V/V); 12.1 min, water/acetonitrile (95:5 V/V) and 14.0 min, water/acetonitrile (95:5 V/V). The column temperature was 40°C, and the injection volume was 2 μL.

MS conditions were set as follows: Electrospray ionisation temperature was set at 500°C. MS voltage was 5,500 V positive and −4,500 V negative. Ion source gas I (GS I) was 55 psi. Gas II (GS II) was 60 psi. The curtain gas was 25 psi. The collision-activated ionization parameter was set to high. Each ion pair was scanned in a triple quadrupole based on the optimized declustering potential and collision energy.

The frozen blood was resuscitated at room temperature, and 1 mL of 80% methanol internal standard extraction agent was added to the samples. The samples were subjected to liquid nitrogen, thawing and vortex three times and centrifuged at 12,000 rpm at 4°C for 10 min. After centrifugation, 200 µL of supernatant was taken into the inner tube of the corresponding bottle for UPLC-MS/MS analysis.

### 2.4 Metabolomics data analysis

The mass spectrum data were processed using the Analyst 1.6.3 software. Principal component analysis (PCA) and orthogonal partial least squares-discriminant analysis (OPLS-DA) were carried out using ropls of R package 3.3.2. OPLS-DA model parameters, R2X, R2Y and Q2, were applied to evaluate model validity. The metabolites were analysed statistically in line with variable importance for the projection (VIP), *P*-value of t-test and fold change (FC). FC ≥ 2, *P* ≤ 0.05 and VIP ≥ 1 were used as criteria for the preliminary screening of metabolites between groups and combined with the Venn diagram, differential metabolites between groups were further screened. MetaboAnalyst 5.0 software (https://www.metaboanalyst.ca/) combined with the Kyoto Encyclopedia of Genes and Genomes (KEGG) and HMDB database was employed to analyse the metabolic pathways of the screened differential metabolites. The sequencing and bioinformatics analysis was performed by Beijing Biomarker Technology.

### 2.5 Statistical analysis

The data were analysed using SPSS 26.0 and presented as the mean ± standard deviation (SD). The *t*-test was utilised to analyse the difference between the two groups, and one-way analysis of variance (ANOVA) was employed to evaluate the statistical significance of exceeding two groups. *P* < 0.05 was considered statistically significant.

## 3 Results

### 3.1 Clinical characteristics of subjects with different vitamin D levels

A total of 15 people with 25(OH)D ≥40 ng/mL (group A), 15 people with 25(OH)D between 30–40 ng/mL (group B) and 10 people with 25(OH)D <30 ng/mL (group C) were enrolled in this study. The clinical information of the subjects recruited is shown in Table 1. There was no remarkable difference among the three groups in age, SBP, DBP, BMI, WC, WHR, TG, TC, HDL, LDL, ALT, AST, GGT, urea, Cr and UA. The 25(OH)D concentration decrease significantly promoted the levels of HbA1c, FBG, FINS, HOMA-IR and TXNIP and inhibited HOMA-β. The levels of HbA1c, FBG, FINS, HOMA-IR and TXNIP were the highest, while HOMA-β was the lowest in the serum of people with 25(OH)D <30 ng/mL. However, the opposite results were observed in the serum of people with 25(OH)D ≥40 ng/mL. The results indicated that vitamin D deficiency led to FBG elevation and insulin resistance.

**Table 1:** Demographic and clinical information of participants

Characteristics	A (*n* = 15)	B (*n* = 15)	C (*n* = 10)	*F*	*P* value
Age (year)	44.93 ± 8.67	43.47 ± 6.66	44.90 ± 8.72	0.155	0.857
SBP (mmHg)	118.40 ± 7.42	118.40 ± 10.11	121.90 ± 10.71	0.526	0.596
DBP (mmHg)	75.00 ± 9.35	73.07 ± 8.15	75.00 ± 6.13	0.26	0.772
BMI (kg/m^2^)	23.90 ± 3.60	23.42 ± 2.30	25.61 ± 3.75	1.455	0.246
WC (cm)	82.07 ± 10.07	78.93 ± 6.72	88.00 ± 9.31	3.237	0.051
WHR (cm/cm)	0.86 ± 0.07	0.82 ± 0.05	0.88 ± 0.03	3.001	0.062
TG (mmol/L)	2.01 ± 1.19	2.77 ± 3.98	2.29 ± 0.97	0.173	0.842
TC (mmol/L)	4.92 ± 1.07	5.35 ± 2.42	5.60 ± 1.29	0.49	0.617
HDL (mmol/L)	1.36 ± 0.30	1.33 ± 0.27	1.29 ± 0.17	0.233	0.793
LDL (mmol/L)	2.71 ± 0.78	3.33 ± 1.73	3.33 ± 0.96	1.224	0.306
ALT (μmol/L)	23.10 ± 7.13	23.55 ± 6.44	25.78 ± 4.53	0.724	0.501
AST (μmol/L)	25.57 ± 6.04	23.55 ± 6.44	25.78 ± 4.53	0.606	0.551
GGT (μ/L)	32.44 ± 17.06	33.72 ± 17.63	62.94 ± 76.03	1.379	0.264
UREA (mmol/L)	4.68 ± 0.99	4.50 ± 1.16	4.81 ± 1.15	0.252	0.779
Cr (mmol/L)	64.05 ± 12.05	58.65 ± 13.34	61.26 ± 12.56	0.796	0.459
UA (mmol/L)	334.61 ± 92.88	281.70 ± 95.99	309.43 ± 96.08	0.451	0.718
HbA1c (ng/mL)	172.17 ± 31.88	210.56 ± 30.34	247.04 ± 19.76	20.721	<0.01
FBG (mmol/L)	5.44 ± 0.66	7.00 ± 2.98	10.46 ± 3.10	16.477	<0.01
FINS (mIU/L)	4.99 ± 0.69	5.75 ± 0.71	6.43 ± 0.62	13.66	<0.01
HOMA-IR	1.22 ± 0.30	1.84 ± 1.00	3.04 ± 1.08	18.212	<0.01
HOMA-β	56.62 ± 18.83	44.48 ± 18.90	22.97 ± 11.67	14.912	<0.01
25(OH)D (ng/mL)	49.12 ± 6.64	36.10 ± 3.72	22.21 ± 3.83	86.09	<0.01
TXNIP (ng/mL)	3.45 ± 0.81	4.59 ± 1.13	6.11 ± 1.16	20.097	<0.01

The data are represented as the mean ± SD. The one-way ANOVA (*F*-test) was utilised to determine differences in various indicators among A, B and C groups. *F* and *P* represented the results of ANOVA analysis among all the groups. A group: people with 25(OH)D ≥40 ng/mL; B group: people with 30 ng/mL ≤ 25(OH)D <40 ng/mL; C group: people with 25(OH)D <30 ng/mL; SBP: systolic blood pressure; DBP: diastolic blood pressure; BMI: body mass index; WC: waist circumference; WHR: waist-hip rate; TG: triglyceride; TC: total cholesterol; HDL: high-density lipoprotein; LDL: low-density lipoprotein; ALT: alanine aminotransferase; AST: aspartate aminotransferase; GGT: gamma-glutamyl transpeptidase; Cr: creatinine; UA: uric acid; HbA1c: haemoglobin A1c; FBG: fasting blood glucose; FINS: fasting insulin; HOMA-IR: homeostasis model assessment of insulin resistance; HOMA-β: homeostasis model assessment-β; 25(OH)D: 25-hydroxyvitamin D; TXNIP: thioredoxin interaction protein.

### 3.2 Analysis of abnormal glucose metabolism in individuals with low vitamin D

The analysis of abnormal glucose metabolism is shown in Table 2. In the A group, people with less than 5.6 mmol/L of FBG accounted for 53.33%, and people with 5.6–7 mmol/L of FBG accounted for 46.67%. In the B group, people with less than 5.6 mmol/L of FBG accounted for 13.33%, people with 5.6–7 mmol/L of FBG accounted for 66.67%, and people with more than 7 mmol/L of FBG accounted for 20%. In the C group, people with 5.6–7 mmol/L of FBG accounted for 30%, people with more than 7 mmol/L of FBG accounted for 70%. There were statistically significant differences among the three groups. Overall, 46.67% of people with 25(OH)D ≥40 ng/mL were diagnosed with prediabetes, 86.67% of people with 30–40 ng/mL of 25(OH)D were diagnosed with prediabetes or diabetes, and all people with 25 (OH)D <30 ng/mL were diagnosed with prediabetes or diabetes. Together, vitamin D deficiency contributes to abnormal glucose metabolism.

**Table 2:** The proportion of abnormal glucose metabolism at different 25(OH)D levels

group	*n*	FBG <5.6 mmol/L (%)	5.6 mmol/L ≤ FBG <7.0 mmol/L (%)	FBG ≥7.0 mmol/L (%)	{\chi }^{2}]	*P*
A	15	8 (53.33%)	7 (46.67%)	0 (0.00%)		
B	15	2 (13.33%)	10 (66.67%)	3 (20.00%)	21.80	<0.001
C	10	0 (0.00%)	3 (30.00%)	7 (70.00%)		

### 3.3 Multivariate analysis of serum metabolites

**Figure 1:**
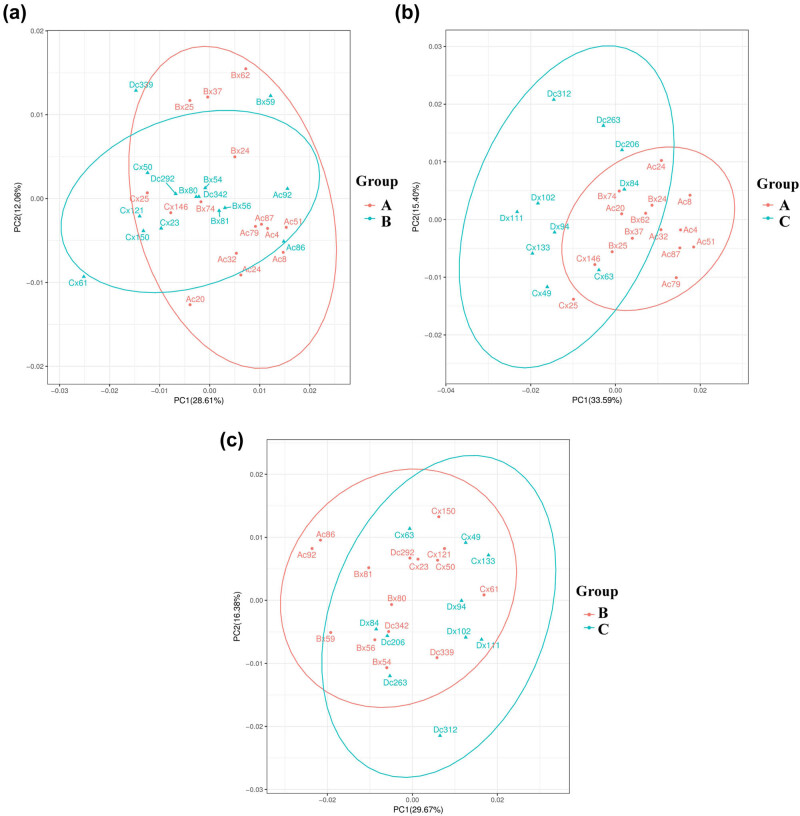
PCA score plots of serum samples from the A, B and C groups. (a) PCA score plots in the B vs A group. (b) PCA score plots in the C vs A group. (c) PCA score plots in the C vs B group. A: people with 25-hydroxyvitamin D (25(OH)D) ≥40 ng/mL; B: people with 30 ng/mL ≤ 25(OH)D <40 ng/mL; C: people with 25(OH)D <30 ng/mL.

**Figure 2:**
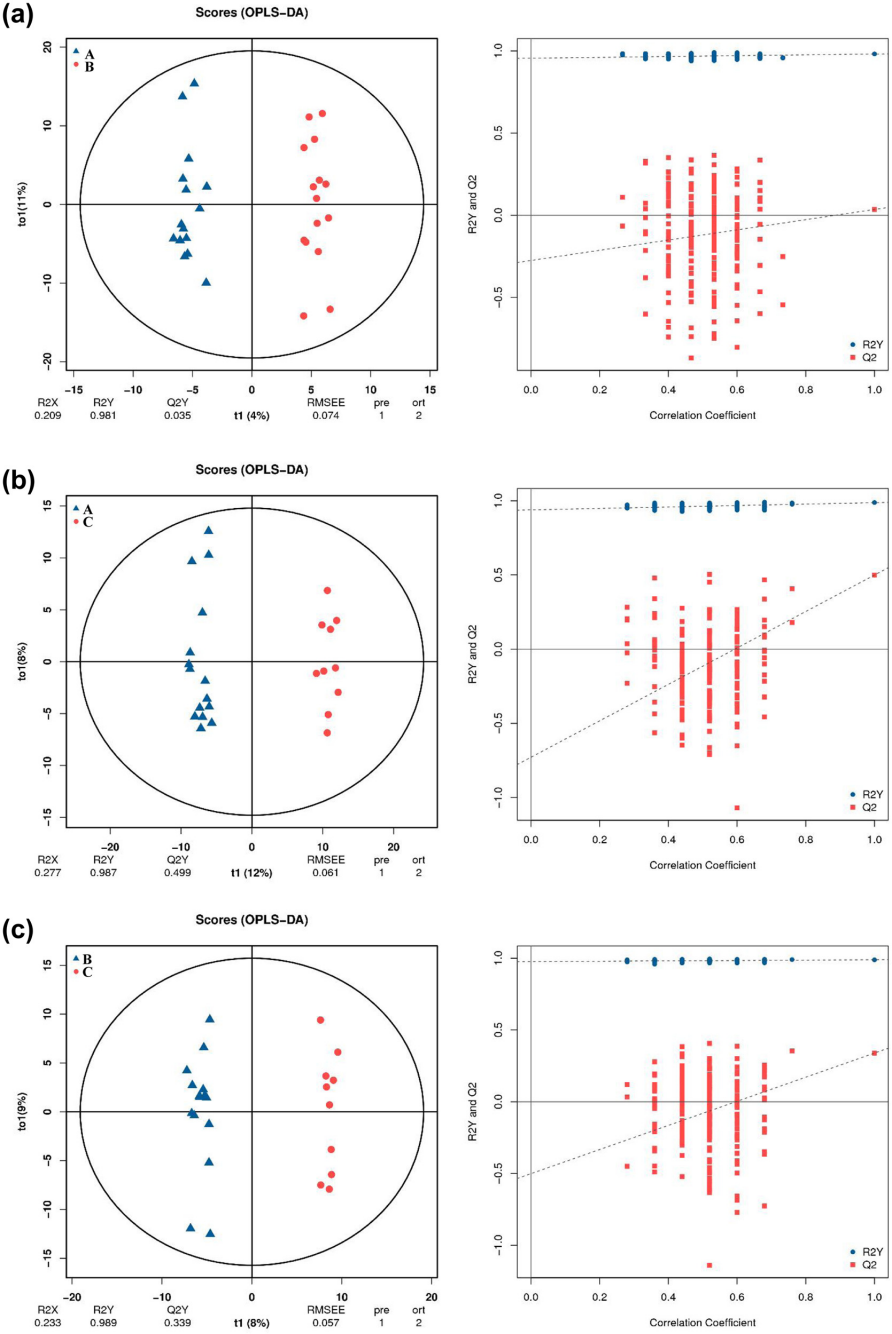
OPLS-DA score plots and OPLS-DA permutation test of serum metabolic profiling. (a) OPLS-DA score plots and OPLS-DA permutation test in B vs A. (b) OPLS-DA score plots and OPLS-DA permutation test in C vs A. (c) OPLS-DA score plots and OPLS-DA permutation test in C vs B.

PCA is an unsupervised analysis method employed to observe the overall distribution trend among samples. The B and A groups were mostly overlapping and isolated in part, indicating that the serum components separation effect of the two groups was not very remarkable (Figure 1a). Similarly, the C vs A and C vs B groups showed the same results (Figure 1b and c). However, given the complexity of metabolomics, PCA analysis may not be capable of differentiating samples of different groups. Thus, to optimise the difference, OPLS-DA was performed. OPLS-DA is a supervised pattern recognition method which can reduce the differences within samples and more accurately characterise the characteristics between samples. OPLS-DA models showed apparent isolation of the metabolic profiles in B vs A, C vs A and C vs B groups (Figure 2a–c). R2Y values in the B vs A, C vs A and C vs B groups were 0.981, 0.987 and 0.989, respectively, suggesting that the model has a good distinction between the samples and that the samples within the same group are highly aggregated. OPLS-DA permutation verification showed that the original R2 and Q2 of the three groups were greater than the corresponding values after Y substitution, suggesting that the three groups were not over-fitted and could be used for the subsequent identification of metabolites. PCA and OPLS-DA score plots revealed a remarkable separation trend in serum comparison groups, indicating that vitamin D affected metabolic profiles.

### 3.4 Identification of potential metabolites related to vitamin D

**Figure 3:**
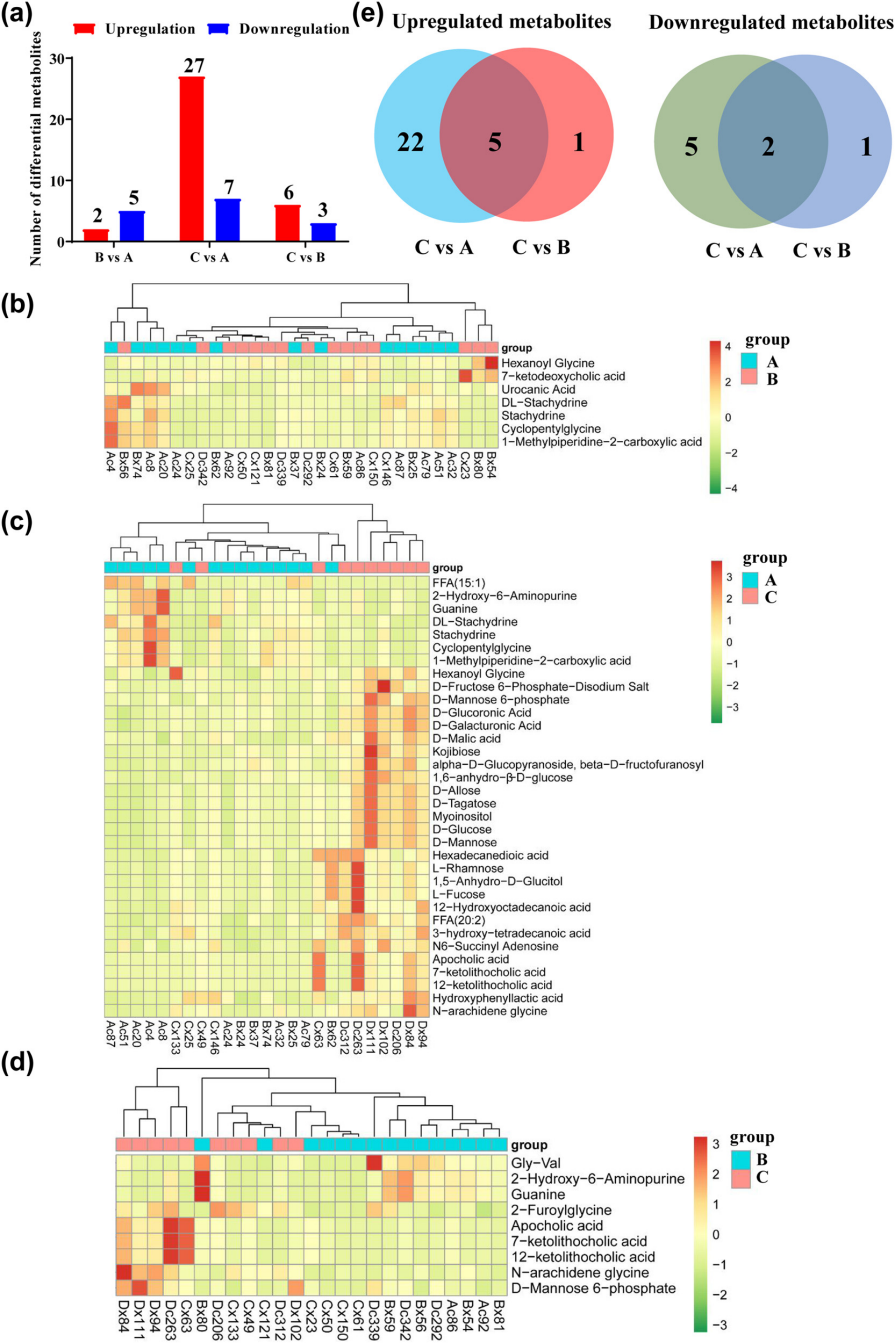
Analyses of differential metabolites in people with different concentrations of vitamin D. (a) The number of differentially upregulated and downregulated metabolites in B vs A, C vs A, and C vs B groups. (b) The heatmap of the metabolites in the B vs A group. (c) The heatmap of the metabolites in the C vs A group. (d) The heatmap of the metabolites in the C vs B group. (e) Venn diagram comparison of upregulated and downregulated metabolites between the C vs A and C vs B groups.

The VIP and *P* values were used to reveal the importance of metabolites. Differential metabolites were screened based on the standard with VIP > 1 and *P* < 0.05. Of 38 differential metabolites, seven, 34, and nine were significantly altered in the B vs A, C vs A and C vs B groups, respectively (Figure 3a, Table 3). Compared with the A group, two metabolites were upregulated in the B group, including hexanoyl glycine and 7-ketodeoxycholic acid, while five were downregulated, including urocanic acid, dl-stachydrine, stachydrine, cyclopentylglycine and 1-methylpiperidine-2-carboxylic acid (Figure 3b). Compared with the A group, 27 metabolites were upregulated in the C group, including 7-ketolithocholic acid, 12-ketolithocholic acid, apocholic acid, d-fructose 6-phosphate-disodium salt, and d-mannose 6-phosphate, while seven metabolites were downregulated, including guanine, 2-hydroxy-6-aminopurine, dl-stachydrine, stachydrine and 1-methylpiperidine-2-carboxylic acid (Figure 3c). Compared with the B group, six metabolites were upregulated in the C group, including *N*-arachidene glycine, 7-ketolithocholic acid, 12-ketolithocholic acid, apocholic acid, d-mannose 6-phosphate and 2-furoylglycine, while three metabolites were downregulated, including Gly-Val, 2-hydroxy-6-aminopurine, and guanine (Figure 3d). Interestingly, there were five common upregulated metabolites and two common downregulated metabolites between the C vs A and C vs B groups (Figure 3e). *N*-arachidene glycine, 7-ketolithocholic acid, 12-ketolithocholic acid, apocholic acid and d-mannose 6-phosphate were upregulated, while 2-hydroxy-6-aminopurine and guanine were downregulated in the C group compared with the A or B groups (Table 3).

**Table 3:** Identification results of differential metabolites in B vs A, C vs A, and C vs B groups in serum

B vs A	Name	A_Mean	B_Mean	Fold_change	log2FC	*P* value	VIP	Regulated
	Hexanoyl glycine	1.31 × 10^−6^	2.63 × 10^−6^	2.005967	0.844079	0.040607	1.905295	Up
	7-Ketodeoxycholic acid	3.86 × 10^−7^	9.76 × 10^−7^	2.529795	1.389847	0.033554	1.98678	Up
	Urocanic acid	1.82 × 10^−5^	8.47 × 10^−6^	0.46546	−0.98821	0.013973	2.055689	Down
	dl-Stachydrine	0.000224	9.33 × 10^−5^	0.41736	−1.62124	0.037106	1.935163	Down
	Stachydrine	0.001707	0.000579	0.339247	−1.44771	0.006879	2.3834	Down
	Cyclopentylglycine	0.00018	8.63 × 10^−5^	0.480418	−0.94127	0.019105	2.125388	Down
	1-Methylpiperidine-2-carboxylic acid	0.00018	8.63 × 10^−5^	0.480418	−0.94127	0.019105	2.125388	Down

C vs A	Name	A_Mean	C_Mean	Fold_change	log2FC	*P* value	VIP	Regulated
	Hexanoyl glycine	1.31 × 10^−6^	2.82 × 10^−6^	2.151301	1.161114	0.007712	1.692944	Up
	1,5-Anhydro-d-glucitol	1.71 × 10^−5^	3.77 × 10^−5^	2.200946	1.230686	0.039567	1.378615	Up
	d-Glucose	9.78 × 10^−6^	1.96 × 10^−5^	2.001923	0.970512	0.001199	2.06755	Up
	d-Mannose	9.78 × 10^−6^	1.96 × 10^−5^	2.001923	0.970512	0.001199	2.06755	Up
	l-Fucose	1.71 × 10^−5^	3.77 × 10^−5^	2.200946	1.230686	0.039567	1.378615	Up
	l-Rhamnose	1.71 × 10^−5^	3.77 × 10^−5^	2.200946	1.230686	0.039567	1.378615	Up
	d-Glucoronic acid	7.13 × 10^−6^	1.75 × 10^−5^	2.460647	1.27646	0.00165	2.084885	Up
	FFA(20:2)	1.18 × 10^−6^	2.36 × 10^−6^	2.00392	1.051267	0.001095	1.93024	Up
	N6-Succinyl adenosine	1.18 × 10^−6^	2.44 × 10^−6^	2.074618	1.116866	0.001011	1.987407	Up
	d-Fructose 6-phosphate-disodium salt	2.89 × 10^−6^	9.52 × 10^−6^	3.297107	1.45351	0.017558	1.666147	Up
	Hydroxyphenyllactic acid	3.99 × 10^−6^	8.15 × 10^−6^	2.042949	1.235163	0.003343	1.714463	Up
	d-Malic acid	1.8 × 10^−6^	4 × 10^−6^	2.226528	1.228234	0.002488	1.850066	Up
	Hexadecanedioic acid	0.000104	0.000219	2.101325	1.139583	0.026595	1.460663	Up
	Myoinositol	9.78 × 10^−6^	1.96 × 10^−5^	2.001923	0.970512	0.001199	2.06755	Up
	N-Arachidene glycine	6.95 × 10^−7^	1.72 × 10^−6^	2.474231	1.256188	0.007601	1.818215	Up
	7-Ketolithocholic acid	2.05 × 10^−5^	0.000211	10.33455	2.534241	0.016816	1.79233	Up
	1,6-Anhydro-β-d-glucose	2.2 × 10^−6^	5.55 × 10^−6^	2.524028	1.228959	0.003908	1.913047	Up
	12-Ketolithocholic acid	2.05 × 10^−5^	0.000211	10.33455	2.534241	0.016816	1.79233	Up
	Apocholic acid	2.05 × 10^−5^	0.000211	10.33455	2.534241	0.016816	1.79233	Up
	d-Mannose 6-phosphate	5.2 × 10^−6^	1.44 × 10^−5^	2.769348	1.285293	0.011309	1.708365	Up
	12-Hydroxyoctadecanoic acid	6.16 × 10^−6^	1.44 × 10^−5^	2.344905	1.189824	0.008935	1.856682	Up
	d-Galacturonic Acid	7.13 × 10^−6^	1.75 × 10^−5^	2.460647	1.27646	0.00165	2.084885	Up
	d-Tagatose	9.78 × 10^−6^	1.96 × 10^−5^	2.001923	0.970512	0.001199	2.06755	Up
	3-Hydroxy-tetradecanoic acid	2.64 × 10^−5^	5.55 × 10^−5^	2.102486	1.183012	0.000731	1.953677	Up
	d-Allose	9.78 × 10^−6^	1.96 × 10^−5^	2.001923	0.970512	0.001199	2.06755	Up
	Kojibiose	2.1 × 10^−5^	5.83 × 10^−5^	2.783261	1.278691	0.019954	1.563984	Up
	alpha-d-Glucopyranoside, beta-d-fructofuranosyl	7.07 × 10^−6^	1.72 × 10^−5^	2.426247	1.217379	0.027871	1.445197	Up
	FFA(15:1)	0.000521	5.16 × 10^−5^	0.098985	−2.38714	0.002171	1.516013	Down
	2-Hydroxy-6-aminopurine	3.14 × 10^−6^	8.59 × 10^−7^	0.273076	−2.29488	0.003089	1.397046	Down
	dl-Stachydrine	0.000224	7.02 × 10^−5^	0.313853	−1.66445	0.00328	1.432255	Down
	Guanine	3.14 × 10^−6^	8.59 × 10^−7^	0.273076	−2.29488	0.003089	1.397046	Down
	Stachydrine	0.001707	0.000566	0.331413	−1.62896	0.00725	1.344179	Down
	Cyclopentylglycine	0.00018	8.12 × 10^−5^	0.45202	−0.841	0.009717	1.290841	Down
	1-Methylpiperidine-2-carboxylic acid	0.00018	8.12 × 10^−5^	0.45202	−0.841	0.009717	1.290841	Down

C vs B	Name	B_Mean	C_Mean	Fold_change	log2FC	*P* value	VIP	Regulated
	N-Arachidene glycine	6.96 × 10^−7^	1.72 × 10^−6^	2.471037	1.177458	0.007508	2.355552	Up
	7-Ketolithocholic acid	3.75 × 10^−5^	0.000211	5.633176	1.875363	0.026005	2.005959	Up
	12-Ketolithocholic acid	3.75 × 10^−5^	0.000211	5.633176	1.875363	0.026005	2.005959	Up
	Apocholic acid	3.75 × 10^−5^	0.000211	5.633176	1.875363	0.026005	2.005959	Up
	d-Mannose 6-phosphate	6.38 × 10^−6^	1.44 × 10^−5^	2.257515	0.996863	0.022986	2.014334	Up
	2-Furoylglycine	3.49 × 10^−6^	7.17 × 10^−6^	2.056719	1.132398	0.00245	2.216046	Up
	Gly-Val	5.12 × 10^−6^	2.15 × 10^−6^	0.420746	−0.50897	0.042666	1.278769	Down
	2-Hydroxy-6-aminopurine	2.49 × 10^−6^	8.59 × 10^−7^	0.344707	−1.4607	0.031704	1.483084	Down
	Guanine	2.49 × 10^−6^	8.59 × 10^−7^	0.344707	−1.4607	0.031704	1.483084	Down

### 3.5 Metabolic pathway analysis of potential metabolites

**Figure 4:**
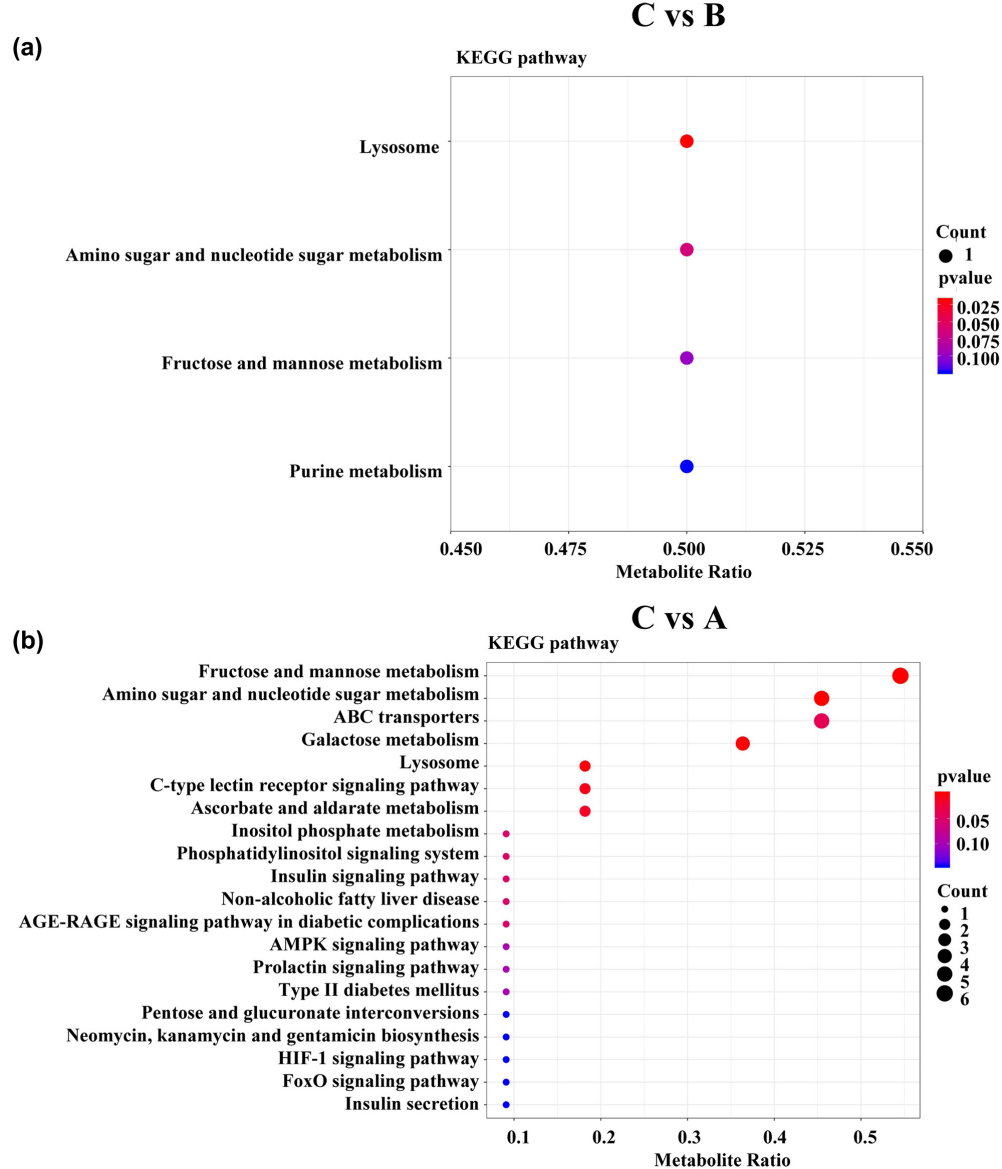
Analyses of metabolic pathways. (a) KEGG pathway analyses of the differential metabolites in C vs B. (b) KEGG pathway analyses of the differential metabolites in C vs A. Each point represents one metabolic pathway. The size of the dots indicates the number of metabolites. Circle colours indicate pathway enrichment significance.

Metabolic pathway enrichment analysis was carried out using the KEGG database. Due to the deficiency of vitamin D in the C group, we focused on the metabolic pathways in the C vs B and C vs A groups. Differentially expressed metabolites in the C vs B group were mainly involved in the lysosome, amino sugar and nucleotide sugar metabolism, fructose and mannose metabolism and purine metabolism (Figure 4a). Differentially expressed metabolites in the C vs A group were mainly related to fructose and mannose metabolism, amino sugar and nucleotide sugar metabolism, ABC transporters and galactose metabolism (Figure 4b).

## 4 Discussion

Vitamin D has a variety of biological functions, including anti-oxidation, anti-inflammation, blood pressure control, immune regulation, apoptosis inhibition and anti-angiogenesis [17,18,19,20]. Some studies demonstrated that 25(OH)D can be significantly reduced in pre-type 2 diabetes mellitus, and the regulation of 1,25(OH)_2_D3 on oxidative stress is dependent on plasma glucose concentration [21,22]. In this study, we found that 46.67% of people with 25(OH)D ≥40 ng/mL were diagnosed with prediabetes, 86.67% of people with 30–40 ng/mL of 25(OH)D were diagnosed with prediabetes or diabetes, and all people with 25(OH)D <30 ng/mL were diagnosed with prediabetes or diabetes. At the same time, with the decrease of 25(OH)D concentration, HbA1c, FBG, FINS, TXNIP and HOMA-IR were increased, whereas HOMA-β was decreased. These results confirm that vitamin D reduction was closely related to blood glucose disorder, pancreatic β cell secretion disorder and insulin resistance.

In the present study, we found that the levels of TC and LDL were increased with a decrease in 25(OH)D concentration. Cholesterol is essential to all cell membranes and exists inside and outside cells. Lipoprotein particles transport cholesterol and other non-polar substances in plasma [23]. LDL consists of cholesterol, protein and phospholipid shell. It is the leading carrier of cholesterol in peripheral tissues. Its components are easily oxidised to produce oxidised LDL [24]. 7-Ketone cholesterol is the most abundant oxysterol in oxidised LDL, which can repress the rate-limiting step in bile acid biosynthesis and strongly suppress the rate-limiting enzyme HMG-CoA reductase in cholesterol biosynthesis [25,26,27]. It is an endogenous regulator of cholesterol biosynthesis. These results suggested that the 25(OH)D concentration decrease was closely related to cholesterol metabolism disorder.

In this study, we discovered that the expression of bile acid metabolites, such as 7-ketodeoxycholic acid, 7-ketolithocholic acid, 12-ketolithocholic acid and apocholic acid, was significantly upregulated when 25(OH)D was less than 30 ng/mL. Bile acids are synthesised by cholesterol in liver parenchymal cells through a classical pathway intervened by cholesterol 7α -hydroxylase (CYP7A1) and an alternate pathway intervened by sterol 27-hydroxylase (CYP27A1). At least 75% of bile acids are produced by the classical pathway under normal conditions. Deoxycholic acid and chenodeoxycholic acid strongly induce 7α- and 7β-hydroxysteroid dehydrogenase to mediate bile metabolism [28]. Elevated lithocholic acid levels during cholestasis were thought to cause liver damage by inducing apoptotic cell death [29]. Over-physiological dose of lithocholic acid can lead to oxidative stress and DNA damage and induce apoptosis of hepatocytes and colonic epithelial cells. Apocholic acid is a dehydrated product of cholic acid and has carcinogenic activity [30]. As a ligand of G protein-coupled receptor, such as TGR5, cholic acid can regulate its synthesis and hepatoenteric recycling and the homeostasis of TG, cholesterol, energy and glucose [31]. Our findings suggested that the disorder of glucose and lipid metabolism and insulin resistance made the bile acid metabolism in the liver overload and further aggravated the oxidative stress of the liver.

Primary bile acids produced by the liver combine with glycine to form conjugated bile acids [32]. In our study, we discovered that hexanoyl glycine, *N*-arachidene glycine, apocholic acid, 2-furoylglycine and d-mannose-6-phosphate were significantly upregulated, and cyclopentylglycine, Gly-Val and 2-hydroxy-6-aminopurine were significantly downregulated when 25(OH)D was under 30 ng/mL, which were closely associated with bile acid metabolism. The abnormal expression of these glycine derivatives was closely related to cholesterol metabolism.

Cholesterol can be oxidised in the skin to 7-dehydrocholesterol (7DHC), which is converted to vitamin D3 by ultraviolet irradiation. Ultraviolet irradiation reduced the expression of 7-dehydrocholesterol reductase (DHCR7), which converted 7DHC into cholesterol. CYP27A1, CYP2R1 and CYP27B1 are critical enzymes in active vitamin D synthesis. Ultraviolet irradiation could significantly upregulate CYP27B1 and promote the synthesis of 1α,25(OH)2D3 [33]. CYP2R1 mRNA dramatically declined in the liver of mice fed with high-fat diet [34]. Therefore, the decrease in 25(OH)VD levels in people with abnormal glucose metabolism in our study may be related to the upregulation of DHCR7 and downregulation of CYP27B1 in the body under the condition of excessive energy load, which leads to vitamin D synthesis disorder and further aggravates oxidative stress and chronic inflammation.

The current study had several limitations. We just analysed the clinical characteristics and compared the differential metabolites and related pathways in people with different vitamin D levels using UPLC-MS/MS. We discovered that the disorder of vitamin D metabolism might be associated with cholesterol metabolism and bile acid biosynthesis. However, there is a lack of validation of differential metabolites involved in these processes. In addition, the potential mechanism of abnormal vitamin D metabolism was not further investigated via *in vitro* and *in vivo* experiments. Our findings can guide future research in abnormal vitamin D metabolism.

In conclusion, we performed a serum metabolic profiling study in people with different levels of vitamin D. A total of seven, 34 and nine differential metabolites were identified in B vs A, C vs A and C vs B groups, respectively. Moreover, 7-ketolithocholic acid, 12-ketolithocholic acid, apocholic acid, *N*-arachidene glycine and d-mannose 6-phosphate were associated with cholesterol metabolism and bile acid biosynthesis. This study will lay the foundation for further research on vitamin D metabolism.

## References

[1] Kim HK, Chung HJ, Le HG, Na BK, Cho MC. Serum 24,25-dihydroxyvitamin D level in general Korean population and its relationship with other vitamin D biomarkers. PLoS One. 2021;16:e0246541. 10.1371/journal.pone.0246541 PMC7894912 33606762

[2] Holick MF, Binkley NC, Bischoff-Ferrari HA, Gordon CM, Hanley DA, Heaney RP, et al. Evaluation, treatment, and prevention of vitamin D deficiency: an Endocrine Society clinical practice guideline. J Clin Endocrinol Metab. 2011;96:1911–30. 10.1210/jc.2011-0385 21646368

[3] Walsh JS, Bowles S, Evans AL. Vitamin D in obesity. Curr Opin Endocrinol Diabetes Obes. 2017;24:389–94. 10.1097/MED.0000000000000371 28915134

[4] Barchetta I, Cimini FA, Cavallo MG. Vitamin D supplementation and non-alcoholic fatty liver disease: present and future. Nutrients. 2017;9(9):1015. 10.3390/nu9091015 PMC5622775 28906453

[5] Fazelian S, Amani R, Paknahad Z, Kheiri S, Khajehali L. Effect of vitamin D supplement on mood status and inflammation in vitamin D deficient type 2 diabetic women with anxiety: A randomized clinical trial. Int J Prev Med. 2019;10:17. 10.4103/ijpvm.IJPVM_174_18 PMC6390422 30820304

[6] Omidian M, Mahmoudi M, Abshirini M, Eshraghian MR, Javanbakht MH, Zarei M, et al. Effects of vitamin D supplementation on depressive symptoms in type 2 diabetes mellitus patients: Randomized placebo-controlled double-blind clinical trial. Diabetes Metab Syndr. 2019;13:2375–80. 10.1016/j.dsx.2019.06.011 31405646

[7] Razzaque MS. Can adverse effects of excessive vitamin D supplementation occur without developing hypervitaminosis D? J Steroid Biochem Mol Biol. 2018;180:81–6. 10.1016/j.jsbmb.2017.07.006 28734988

[8] Tao YL, Tang XL, Guan CH. Advances in the effects of supplementation vitamin D on metabolic syndrome. J Hainan Med Univ. 2021;27:396–400.

[9] Al-Agha AE, Alafif MM, Abd-Elhameed IA. Glycemic control, complications, and associated autoimmune diseases in children and adolescents with type 1 diabetes in Jeddah, Saudi Arabia. Saudi Med J. 2015;36:26–31. 10.15537/smj.2015.1.9829 PMC4362194 25630001

[10] Kostoglou-Athanassiou I, Athanassiou P, Gkountouvas A, Kaldrymides P. Vitamin D and glycemic control in diabetes mellitus type 2. Ther Adv Endocrinol Metab. 2013;4:122–8. 10.1177/2042018813501189 PMC3755528 23997931

[11] Sung CC, Liao MT, Lu KC, Wu CC. Role of vitamin D in insulin resistance. J Biomed Biotechnol. 2012;2012:634195. 10.1155/2012/634195 PMC3440067 22988423

[12] Kongsbak M, Levring TB, Geisler C, von Essen MR. The vitamin d receptor and T cell function. Front Immunol. 2013;4:148. 10.3389/fimmu.2013.00148 PMC3684798 23785369

[13] Xie G, Wang L, Chen T, Zhou K, Zhang Z, Li J, et al. A metabolite array technology for precision medicine. Anal Chem. 2021;93:5709–17. 10.1021/acs.analchem.0c04686 33797874

[14] Rodriguez-Morato J, Pozo OJ, Marcos J. Targeting human urinary metabolome by LC-MS/MS: a review. Bioanalysis. 2018;10:489–516. 10.4155/bio-2017-0285 29561651

[15] Huang R, Cathey S, Pollard L, Wood T. UPLC-MS/MS analysis of urinary free oligosaccharides for lysosomal storage diseases: Diagnosis and potential treatment monitoring. Clin Chem. 2018;64:1772–9. 10.1373/clinchem.2018.289645 30201803

[16] Ly TK, Ho TD, Behra P, Nhu-Trang TT. Determination of 400 pesticide residues in green tea leaves by UPLC-MS/MS and GC-MS/MS combined with QuEChERS extraction and mixed-mode SPE clean-up method. Food Chem. 2020;326:126928. 10.1016/j.foodchem.2020.126928 32408000

[17] Plum LA, DeLuca HF. Vitamin D, disease and therapeutic opportunities. Nat Rev Drug Discov. 2010;9:941–55. 10.1038/nrd3318 21119732

[18] Chagas CE, Borges MC, Martini LA, Rogero MM. Focus on vitamin D, inflammation and type 2 diabetes. Nutrients. 2012;4:52–67. 10.3390/nu4010052 PMC3277101 22347618

[19] Guillot X, Semerano L, Saidenberg-Kermanac’h N, Falgarone G, Boissier MC. Vitamin D and inflammation. Jt Bone Spine. 2010;77:552–7. 10.1016/j.jbspin.2010.09.018 21067953

[20] Tarcin O, Yavuz DG, Ozben B, Telli A, Ogunc AV, Yuksel M, et al. Effect of vitamin D deficiency and replacement on endothelial function in asymptomatic subjects. J Clin Endocrinol Metab. 2009;94:4023–30. 10.1210/jc.2008-1212 19584181

[21] Xie X, Bai G, Liu H, Zhang L, He Y, Qiang D, et al. Early predictors in the onset of type 2 diabetes at different fasting blood glucose levels. Diabetes Metab Syndr Obes. 2021;14:1485–92. 10.2147/DMSO.S301352 PMC8020326 33833539

[22] Abu El Maaty MA, Almouhanna F, Wolfl S. Expression of TXNIP in cancer cells and regulation by 1,25(OH)(2)D(3): Is it really the Vitamin D(3) upregulated protein? Int J Mol Sci. 2018;19:796. 10.3390/ijms19030796 PMC5877657 29534438

[23] Cox RA, Garcia-Palmieri MR. Cholesterol, triglycerides, and associated lipoproteins. In: Walker HK, Hall WD, Hurst JW, editors. Clinical Methods: The History, Physical, and Laboratory Examinations. Boston: Butterworths; 1990. 21250045

[24] Dias HK, Brown CL, Polidori MC, Lip GY, Griffiths HR. LDL-lipids from patients with hypercholesterolaemia and Alzheimer’s disease are inflammatory to microvascular endothelial cells: mitigation by statin intervention. Clin Sci (Lond). 2015;129:1195–206. 10.1042/CS20150351 PMC5055810 26399707

[25] Oh MJ, Zhang C, LeMaster E, Adamos C, Berdyshev E, Bogachkov Y, et al. Oxidized LDL signals through Rho-GTPase to induce endothelial cell stiffening and promote capillary formation. J Lipid Res. 2016;57:791–808. 10.1194/jlr.M062539 PMC4847627 26989083

[26] Lyons MA, Brown AJ. 7-Ketocholesterol. Int J Biochem Cell Biol. 1999;31:369–75. 10.1016/s1357-2725(98)00123-x 10224662

[27] Brown AJ, Dean RT, Jessup W. Free and esterified oxysterol: formation during copper-oxidation of low density lipoprotein and uptake by macrophages. J Lipid Res. 1996;37:320–35. 9026530

[28] Sutherland JD, Macdonald IA. The metabolism of primary, 7-oxo, and 7 beta-hydroxy bile acids by Clostridium absonum. J Lipid Res. 1982;23:726–32. 7119570

[29] Barrasa JI, Olmo N, Lizarbe MA, Turnay J. Bile acids in the colon, from healthy to cytotoxic molecules. Toxicol Vitro. 2013;27:964–77. 10.1016/j.tiv.2012.12.020 23274766

[30] Lacassagne A, Buu-Hoi NP, Zajdela F. Carcinogenic activity of apocholic acid. Nature. 1961;190:1007–8. 10.1038/1901007a0 13831121

[31] Thomas C, Pellicciari R, Pruzanski M, Auwerx J, Schoonjans K. Targeting bile-acid signalling for metabolic diseases. Nat Rev Drug Discov. 2008;7:678–93. 10.1038/nrd2619 18670431

[32] Adeva-Andany M, Souto-Adeva G, Ameneiros-Rodriguez E, Fernandez-Fernandez C, Donapetry-Garcia C, Dominguez-Montero A. Insulin resistance and glycine metabolism in humans. Amino Acids. 2018;50:11–27. 10.1007/s00726-017-2508-0 29094215

[33] Shin MH, Lee Y, Kim MK, Lee DH, Chung JH. UV increases skin-derived 1alpha,25-dihydroxyvitamin D3 production, leading to MMP-1 expression by altering the balance of vitamin D and cholesterol synthesis from 7-dehydrocholesterol. J Steroid Biochem Mol Biol. 2019;195:105449. 10.1016/j.jsbmb.2019.105449 31470109

[34] Roizen JD, Long C, Casella A, O’Lear L, Caplan I, Lai M, et al. Obesity decreases hepatic 25-hydroxylase activity causing low serum 25-hydroxyvitamin D. J Bone Min Res. 2019;34:1068–73. 10.1002/jbmr.3686 PMC6663580 30790351

